# The Role of Patient Involvement When Developing Therapies

**DOI:** 10.1089/nat.2021.0048

**Published:** 2022-04-19

**Authors:** Annemieke Aartsma-Rus, Elizabeth Vroom, Daniel O'Reilly

**Affiliations:** ^1^ Department of Human Genetics, Leiden University Medical Center, Leiden, The Netherlands.; ^2^ Duchenne Parent Project, Veenendaal, The Netherlands.; ^3^ RNA Therapeutics Institute, University of Massachusetts Medical School, Worcester, Massachusetts, USA.

**Keywords:** patient advocates, rare disease, drug development

## Abstract

The drug development process is a long and arduous one, especially for rare diseases. Patient and patient representatives can and should be involved in this process from an early stage, since they have the perspective of living with a disease on a daily basis and can best identify which symptoms are the largest burden and which benefits would be more important to them. In this perspective, we outline how patients can be involved optimally in drug development. We outline success factors such as finding the right partners, bilateral education, having realistic expectations, and an open and honest dialog with all stakeholders.

## Introduction

There are many ways patients and patient representatives can be involved in fundamental and translational research into their disease. They can actively participate by providing samples (DNA, tissues, fluids, etc.) and taking part in natural history studies and clinical trials. They can contribute financially by funding research. However, in an ideal setting, they are also partners in the drug development process. They are best suited to indicate which therapeutic effects they want most from a therapy, which can then be used to develop outcome measures to test clinical benefit in clinical trials. Likewise, their preferences regarding benefit risk should be taken into consideration and they should be involved in discussions about clinical trial design, to, for example, avoid undue burden of the clinical study and to optimize selection of outcome measures.

A multistakeholder approach, where patients, researchers, clinicians, regulators, and industry have an ongoing dialog to jointly identify gaps in knowledge and align future work has been successfully used in the Duchenne muscular dystrophy field [1,2]. Duchenne muscular dystrophy is a severe progressive muscle-wasting disease characterized by the irreversible loss of one motor function after the other [3]. There is a long track record of patient involvement for this disease, as national parent projects were initiated already more than 25 years ago in multiple countries.

However, the question is where and how to start this collaborative approach. In the current perspective, we will outline different ways to initiate networking and how to optimally utilize it for drug development. Using the Duchenne muscular dystrophy example as a paradigm we will outline success factors and lessons learnt that are applicable also to the larger rare disease community. Since N-of-1 therapies are developed for patients with private mutations, we will focus on genetic rare diseases.

## Steps of Therapy Development for Genetic Rare Diseases

**FIG. 1.**
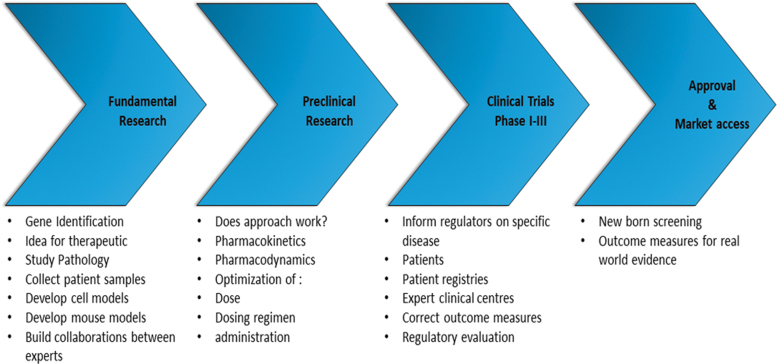
The four key stages of drug development: Fundamental Research, Preclinical Research, Clinical Trials (Phase I-III), and Approval & Market access. Color images are available online.

From a patient perspective, drug development can be an arduous and long process (Fig. 1). Once causative mutations are discovered, the disease pathology can be studied in more detail. Sometimes these fundamental studies will identify potential inroads for therapeutic approaches that can then be evaluated in cell and animal models. Here, it is important to distinguish proof-of-concept studies, showing that the rationale of the drug is correct, and preclinical studies where preparative studies are done toward clinical trials to optimize, for example, dosing, treatment regimen, and route of administration.

Especially for rare diseases with unmet medical need there is a tendency to (partially) skip preclinical studies and move directly from proof-of-concept studies to clinical trials. Many rare diseases, including those in the neuromuscular field, have a poor track record, where potential therapies were “effective” in animal models, but then failed when tested in patients in clinical trials [4]. Often this is used to discredit the use of animal model systems. However, we would argue that in this case, suboptimal use of model systems is at fault. Since one generally only has one chance at a clinical trial for a specific compound in a rare disease, it is imperative to gather as much information as possible on optimal dosing, treatment regimen, etc.

Traditionally clinical trials involve phase 1 studies in healthy volunteers, followed by phase 2 and pivotal phase 3 studies in patients. For genetic therapies healthy volunteer studies will provide limited information, since therapies either only apply to mutated transcripts (which will not be present in healthy volunteers) or have the opposite effect (e.g., for Duchenne muscular dystrophy the exon skipping approach restores dystrophin production in patients but would disrupt it in healthy volunteers). As such, rare disease therapies often start with a phase 1/2 study in patients, assessing first safety and pharmacology and then assessing therapeutic effects in placebo-controlled studies.

If a therapeutic approach is safe and effective, a dossier is submitted to the regulatory agencies, who upon establishing a positive benefit and risk ratio will provide marketing authorization for the therapy. However, discussions with regulatory agencies start far before submission and include for example Protocol Assistance [5], a special form of scientific advice available for developers of designated orphan medicines for rare diseases. Especially for rare diseases with no or limited clinical trial history, initiating a dialog with the regulators early will be mutually beneficial since regulators will become familiar with a specific rare disease and its peculiarities and specifics, while the clinical researchers will become familiar with regulatory procedures. When Market Authorization is granted, the drug will be marketed and efficacy and safety, but also cost-effectiveness will be continuously assessed in postmarketing studies.

It is important to bear in mind that the drug development process critically depends on infrastructure and tools. Fundamental research cannot be done without patient samples; *in vitro* and *in vivo* studies need cell and animal models. For clinical trials, patient registries are useful. Furthermore, clinical trials in rare diseases are often conducted in multiple centers. For this standardizing patient care is crucial as is knowing where centers of expertise are for a particular rare disease. To measure clinical benefit, you need an outcome measure that measures something patients find relevant in a standardized way. To design an optimal clinical trial, you need detailed (and recent) natural history information of the most important outcome measures.

Often this infrastructure is lacking and developed at the same time as the initiation of clinical trials. This is, however, very risky as lacking natural history information, proper power calculations cannot be done. Especially for therapies with a modest therapeutic effect, there is a high risk for false-negative findings: the therapy worked but one was unable to detect this due to suboptimal trial design. Multiple collaborative networks have been initiated to generate the infrastructure for diseases, for example, TREAT-NMD in the neuromuscular field [6] and ENROLL-HD for Huntington's disease [7].

Once drugs are marketed, they will be tested in the “real world.” Real-world evidence can provide the “validation” between the results seen in clinical studies that initially supported approval in a selected group of patients, and the results when a drug is used in a less controlled setting over a longer time. For rare disease, drugs often obtaining sufficient evidence to obtain full marketing authorization is challenging. To ensure earlier access for patients with unmet medical need regulatory systems can also approve drugs based on more limited information, using, for example, conditional marketing authorization (European Medicines Agency) and accelerated approval (Food and Drug Administration USA) mechanisms. Here, still balanced benefit risk profile is needed, but a more limited data package is accepted. However, these approvals come with postmarketing obligations to collect additional data in postmarketing studies.

Many rare diseases have a progressive nature where with time, functions are irreversibly lost. The therapeutic effect will depend on the disease phase when treatment is initiated. Earlier treatment is anticipated to result in larger benefit. New-born screening is a way to facilitate very early treatment initiation. However, adding a disease to existing new-born screening efforts in a country is a long process. In many countries, a disease is currently only included in a new-born screening program when there is a cure available, however, starting the procedure for new-born screening for a given disease in a given country may take years, so by default you are too late when you start once a drug is approved.

## Success Factors Toward Building a Good Multistakeholder Network

### Education

Toward building a multistakeholder network, where patients play a central role, there are several important success factors. The first is education.

In the drug development dialog, patients and patient representatives are needed to provide the patient perspective. While patients are not expected to provide advice on drug mechanisms or statistical models, basic understanding of the drug development process, the disease pathology, and the tools needed to develop therapies are crucial to provide useful feedback. In addition, understanding the perspectives of the other stakeholders and their procedures will be helpful to have constructive discussions.

### Raising awareness

It is important all stakeholders are aware of the challenges and needs patients experience due to their disease. Patient preferences and patient perspective on burden of living with the disease, trial participation, and the burden of the treatment as well as how patients perceive benefits of trial participation and receiving treatments should be part of decision making at different levels. Since different diseases involve different burdens and challenges, one has to involve disease-specific patient representatives in these processes, rather than a token patient representative with an unrelated disease.

### Patient education

There are many options where patients can be educated. One example would be the training opportunities organized by the European Organization for Rare Diseases (EURORDIS), as these focus on all rare diseases. EURORDIS has an open academy where individuals can sign up for free to take online courses on selected topics on health care, research, and medicine development. In-person options include the Summer School, which is on medicine research and development, the Winter School, which is on fundamental and preclinical research, model systems, and genetic analyses. Originally, these were face-to-face trainings, but currently they are run online due to the coronavirus disease 2019 (COVID-19) pandemic.

Global Genes is nonprofit, based in the United States, whose purpose is to connect and inform the global rare disease community and other stakeholders [8]. For rare disease patients and advocates, Global Genes has a RARE University, which provides courses on topics, such as drug development, genetics, and data collection, all aimed at nonscientists. Global Genes also have developed a RARE Foundation Alliance [9], whose aim is to bring together stakeholders, such as industry, academics, and patient advocates to foster networking and collaboration. The Foundation Alliance currently has >750 members.

Global genes also organize symposiums and conferences as means for networking, education, and dissemination, with a RARE Drug Development Symposium and RARE Patient Advocacy summit taking place every year. The RARE Drug Development Symposium, fosters these collaborations with an aim to focus on developing drugs, with a broad range of topics covered helping to guide patient advocates to better understanding the drug development process from preclinical research even up to clinical trials. Topics have included: preclinical models, ethical considerations, and how to approach applying for funding. The RARE Patient Advocacy Summit has a broader set of talks and workshops, which can help patients and advocates learn, gain skills, and provide further networking opportunities.

### Patients educating

At the same time, patients also have to educate the other stakeholders. No one else knows what it is like to live with the disease on a day-to-day basis [10,11]. Learning from patients means that academics develop therapies that address symptoms that are most burdensome to patients and develop outcome measures that measure something that patients find relevant. For example, in the Duchenne field, the first pivotal trials used the 6-min walk test as the primary endpoint. This outcome measure is not developed for Duchenne but was borrowed from the cardiovascular field lacking other outcome measures. As such, it was more difficult to translate findings to clinical benefit as perceived by patients. Furthermore, the measure can only be used in ambulant patients, thus excluding the majority of Duchenne patients. By contrast, the performance upper limb test [12] was developed in collaboration with patients and patient organizations were actively involved in the development [12]. In this study, the development started with asking which arm functions were important to patients. This was taken into account when setting up the test items and therefore losing points on this scale means that patients have lost a function that is important to them.

Education by patients also means that regulators, knowing the burden of having a disease, can better assess benefit risk profile of potential therapies. Finally, for companies, it means they can optimize their clinical trial design taking into account the burdens of the disease and selecting optimal outcome measures.

There are systems in place that can foster these educational dialogs, for example, the European Medicines Agency involves patient representatives when sponsors approach them for protocol assistance. To streamline input from patients to companies, community advisory boards can be set up for diseases or disease groups. These can be part of the EURORDIS program for community advisory boards or be independent and allow a company to present their drug development ideas and clinical trial setups and receive feedback from a group of patient advisors. Patient organizations can also play an active role by performing preference studies. For example, the parent project muscular dystrophy coordinated a preference study on acceptable side effects [13] for therapies slowing down disease progression with carers of Duchenne patients and one on the risk perception [14] for gene therapies with Duchenne patients and carers. These studies also will be informative to researchers, clinicians, and pharmaceutical companies and will allow focusing on symptoms that are more burdensome to patients.

As with most things, optimal communication is crucial to streamline processes and optimally benefit from input of all stakeholders. This involves also learning each other's language and procedures. It is easy to make assumptions about the perceived burden of a disease. However, these assumptions are likely wrong. For example, the quality of life of Duchenne muscular dystrophy patients is scored higher by patients than their parents and caregivers, who again score higher than clinicians [15].

For researchers and clinicians attending patient conferences and webinars is crucial to gain more insight in the day-to-day burden of the disease. Researchers often do not professionally interact with patients, and these conferences are unique opportunity to discuss with patients and caregivers and to learn about their day-to-day struggles. Clinicians will see patient but generally only in the hospital setting.

### How to find the right partners

Discovering researchers and specialists working on a specific disease can be challenging, especially for the ultrarare diseases. Orphanet can be used as a starting point [16]. It provides information on patient organizations, researchers, clinical experts, and expert centers.

When interacting with academics, this should be a dialog. As mentioned, fundamental and preclinical researchers do not professionally interact with patients and therefore may be less aware of the impact symptoms have on daily living as they primarily have textbook knowledge about this. It is also important to bear in mind as patient organizations that academics are often specialist in a specific subfield, and that fundamental researchers may not have knowledge and expertise in translational development, and that most researchers have very little knowledge of the regulatory system. Notably, since 2015, the EURORDIS summer school is also open to researchers, so this is a way translational and clinical researchers can obtain more knowledge about drug approval and postapproval mechanism.

Generally speaking, most academic researchers will be keen to help and interact with patients. When considering funding academics, it is better to fund projects than an individual researcher to ensure that a specific goal is reached. It is important patient organizations have an independent advisory board or a peer review system in place to evaluate these projects. This is helpful to ensure that realistic projects are funded that are more likely to have an impact on the disease than those that overpromise. Since most researchers have a narrow expertise, it is best to select a number of advisors with complementary skill sets.

It is now more and more common that national and international funders request that patients are involved in research projects. Here, it is that researchers ensure that patients and patient representatives can actively contribute in a timely fashion, rather than be a tick box activity to confirm everything is on the right track at a time when it is too late to change the focus (e.g., to a symptom that patients find more relevant).

### How to interact with industry partners

Companies should be discouraged to set up their own patient advisory boards, with handpicked members, but rather get their information from Community Advisory boards set up by patient organizations with independent well-trained patients or patient representatives.

Special attention is needed to the fact that patient (and researchers) involved in dialogs about drug development with companies will have a conflict of interest which makes it difficult to engage with regulatory bodies. The European Medicines Agency has strict conflict of interest rules in place [17,18]. As for most rare diseases and ultra-rare diseases, only a few patient representatives are available, it is important very early in the process the decision will be taken who will be advising companies and who will interact with regulators, to avoid that all patient representative for a given disease become conflicted. For the majority of declared interests, a 3-year cooling-off period is prescribed.

### Having realistic expectations

It is important that patient representatives have realistic expectations from potential therapies. Only then will they be able to properly balance potential side effects and treatment burdens. Sometimes patient representatives have unrealistic expectations with regard to the potential benefit of a therapy but also the timeline of therapy development.

Therapy development is a challenging process, where success in an early step is no guarantee for success in the following steps. Still, many scientific publications describing proof-of-concept studies in model systems for a potential therapy contain promises in the abstract and the concluding remarks that “a treatment for disease x is now on the horizon.” Scientists will know the downstream challenges that still have to be faced and will be able to interpret this properly. However, in the current age, online publications are also accessible to patient groups, and it will be much more difficult for them to put comments like these into context.

Second, there is a tendency to publish primarily on therapies that are effective. There is fortunately a shift with journals, including this one, to also actively inviting authors to submit articles on “negative results.” In this study, the phrase, negative results, holds a negative connotation that is actually not correct. It is part of the scientific process to have a hypothesis and to test it. Knowing that the hypothesis is wrong, also advances science. Furthermore, not publishing on compounds that were tested but did not induce therapeutic effects leads to waste, due to others testing the same compound not knowing it is not effective.

The key to making sure there are realistic expectations, is through open and honest communication with stakeholders about the potential limitations of a therapeutic. Patients can also ask scientists who are not directly involved in the development of a specific drug to give advice based on their expertise.

### N-of-1 treatments

For the development of N-of-1 treatments, direct involvement of patients and carers is crucial. An ongoing dialog between the clinician who will treat the patient and the patient and carers will be key, since the patient or caregiver is the only one who can indicate what would constitute benefit for him/her, but also what would be an unacceptable side effect. These discussions need to take place before the first treatment and start and stop criteria should be discussed regularly and changed if needed. The challenge with N-of-1 treatments is that they are often mutation specific rather than disease specific. Thus, the outcome measures will differ between different patients.

When assessing whether a certain N-of-1 approach, for example, splice modulating antisense oligonucleotides, such as milasen [19], is successful, this would be based on a collection of case studies, making it difficult to draw general conclusions. One possible solution would be to use the goal attainment scale [20]. In this scale each individual indicates a benefit they hope to gain that is then evaluated on a linear scale that can measure both improvement and a decline. Benefit can take many forms, from improving a specific function, maintaining a function, or a slower loss of a function than expected from natural history. While the form of benefit will vary between patients, the level of benefit can now be compared across different diseases.

## Conclusions

In conclusion, we highlight that patient advocates can and should be involved in all the steps of therapy development for rare diseases. A successful partnership relies on several success factors: Patient finding the right stakeholders, and patients educating stakeholders on the true burdens of a disease, which can lead to the development of more accurate therapies. Also, having open and honest conversations about timelines, goals, and limitations of the drug development process between all stakeholders. Our commentary provides information about these factors to all stakeholders to help maximize the positive impact of patients and advocates on research.

## References

[1] Straub V, P Balabanov, K Bushby, M Ensini, N Goemans, A De Luca, A Pereda, R Hemmings, G Campion, *et al.* (2016). Stakeholder cooperation to overcome challenges in orphan medicine development: the example of Duchenne muscular dystrophy. Lancet Neurol 15:882–890. 27302365 10.1016/S1474-4422(16)30035-7

[2] Aartsma-Rus A, E Mercuri, E Vroom and P Balabanov. (2018). Meeting report of the “Regulatory Exchange Matters” session at the 5th International TREAT-NMD Conference:: lessons in communication: how an early dialogue between patients, regulators and academics can further therapy development for neuromuscular disorders Freiburg, Germany, 27–29 November 2017. Neuromuscul Disord 28:619–623. 10.1016/j.nmd.2018.04.009 29778308

[3] Duan D, N Goemans, S i Takeda, E Mercuri and A Aartsma-Rus. (2021). Duchenne muscular dystrophy. Nat Rev Dis Primers 7:13. 33602943 10.1038/s41572-021-00248-3 PMC10557455

[4] Willmann R, J Lee, C Turner, K Nagaraju, A Aartsma-Rus, DJ Wells, KR Wagner, C Csimma, V Straub, MD Grounds and A De Luca. (2020). Improving translatability of preclinical studies for neuromuscular disorders: lessons from the TREAT-NMD Advisory Committee for Therapeutics (TACT). Dis Model Mech 13:dmm042903. 32066568 10.1242/dmm.042903 PMC7044444

[5] EMA. Protocol Assistance. https://www.ema.europa.eu/en/human-regulatory/research-development/scientific-advice-protocol-assistance#protocol-assistance-section2021. Accessed September 6, 2021.

[6] TREAT-NMD. https://treat-nmd.org/. Accessed October 6, 2021.

[7] ENROLL-HD. https://enroll-hd.org/. Accessed June 18, 2021.

[8] Genes G. https://globalgenes.org/#. Accessed October 6, 2021.

[9] Genes G. RARE Foundation Alliance. https://globalgenes.org/foundation-alliance/. Accessed June 20, 2021.

[10] Verhaart IEC, A Johnson, S Thakrar, E Vroom, F De Angelis, F Muntoni, AM Aartsma-Rus and EH Niks. (2019). Muscle biopsies in clinical trials for Duchenne muscular dystrophy—patients' and caregivers' perspective. Neuromuscul Disord 29:576–584. 31378431 10.1016/j.nmd.2019.06.004

[11] Gaasterland CMW, MCJ van der Weide, MJ du Prie-Olthof, M Donk, MM Kaatee, R Kaczmarek, C Lavery, K Leeson-Beevers, N O'Neill, *et al.* (2019). The patient's view on rare disease trial design—a qualitative study. Orphanet J Rare Dis 14:31. 30732630 10.1186/s13023-019-1002-z PMC6367834

[12] Mayhew A, ES Mazzone, M Eagle, T Duong, M Ash, V Decostre, M Vandenhauwe, K Klingels, J Florence, et al.; Performance of the Upper Limb Working Group. (2013). Development of the performance of the upper limb module for Duchenne muscular dystrophy. Dev Med Child Neurol 55:1038–1045. 23902233 10.1111/dmcn.12213

[13] Hollin IL, HL Peay and JF Bridges. (2015). Caregiver preferences for emerging Duchenne muscular dystrophy treatments: a comparison of best-worst scaling and conjoint analysis. Patient 8:19–27. 25523316 10.1007/s40271-014-0104-x

[14] Paquin RS, R Fischer, C Mansfield, B Mange, K Beaverson, A Ganot, AS Martin, C Morris, C Rensch, *et al.* (2019). Priorities when deciding on participation in early-phase gene therapy trials for Duchenne muscular dystrophy: a best–worst scaling experiment in caregivers and adult patients. Orphanet J Rare Dis 14:102. 31072340 10.1186/s13023-019-1069-6 PMC6509771

[15] Bach JR, DI Campagnolo and S Hoeman. (1991). Life satisfaction of individuals with Duchenne muscular dystrophy using long-term mechanical ventilatory support. Am J Phys Med Rehabil 70:129–135. 2039614 10.1097/00002060-199106000-00004

[16] ORPHANET. The Portal for rare disease and oprhan drugs. https://www.orpha.net/consor/cgi-bin/index.php?lng=EN. Accessed January 6, 2021.

[17] EUPATI. Guidance for patient involvement in industry-led medicines R&D. https://toolbox.eupati.eu/resources/guidance-for-patient-involvement-in-industry-led-medicines-rd/. Accessed October 6, 2021. 10.3389/fmed.2018.00270 PMC6190844 30356834

[18] EURODIS. EURODIS Charter. https://download2.eurordis.org.s3-eu-west-1.amazonaws.com/clinical_trials/charter-for-collaboration-in-clinical-research.pdf. Accessed September 6, 2021.

[19] Kim J, C Hu, C Moufawad El Achkar, LE Black, J Douville, A Larson, MK Pendergast, SF Goldkind, EA Lee, *et al.* (2019). Patient-customized oligonucleotide therapy for a rare genetic disease. N Engl J Med 381:1644–1652. 31597037 10.1056/NEJMoa1813279 PMC6961983

[20] Gaasterland CMW, MCJ van der Weide, KCB Roes and JH van der Lee. (2019). Goal attainment scaling as an outcome measure in rare disease trials: a conceptual proposal for validation. BMC Med Res Methodol 19:227. 31801463 10.1186/s12874-019-0866-x PMC6894223

